# *In Silico* Models for Design and Optimization of Science-Based *Listeria* Environmental Monitoring Programs in Fresh-Cut Produce Facilities

**DOI:** 10.1128/AEM.00799-21

**Published:** 2021-10-14

**Authors:** Genevieve Sullivan, Claire Zoellner, Martin Wiedmann, Renata Ivanek

**Affiliations:** a Department of Food Science, College of Agriculture and Life Sciences, Cornell University, Ithaca, New York, USA; b iFoodDecisionSciences, Seattle, Washington, USA; c Department of Population Medicine and Diagnostic Sciences, Cornell University, Ithaca, New York, USA; The Pennsylvania State University

**Keywords:** agent-based modeling, *Listeria*, environmental monitoring, produce

## Abstract

Food facilities need time- and cost-saving methods during the development and optimization of environmental monitoring for pathogens and their surrogates. Rapid virtual experimentation through *in silico* modeling can alleviate the need for extensive real-world, trial-and-error style program design. Two agent-based models of fresh-cut produce facilities were developed as a way to simulate the dynamics of *Listeria* in the built environment by modeling the different surfaces of equipment and employees in a facility as agents. Five sampling schemes at three time points were evaluated *in silico* on their ability to locate the presence of *Listeria* contamination in a facility with sample sites for each scheme (i.e., scenario, as modeled using scenario analysis) based on the following: the facilities’ current environmental monitoring program (scenario 1), Food and Drug Administration recommendations (scenario 2), random selection (scenario 3), sites exclusively from zone 3 (i.e., sites in the production room but not directly adjacent to food contact surfaces) (scenario 4), or model prediction of elevated risk of contamination (scenario 5). Variation was observed between the scenarios on how well the *Listeria* prevalence of the virtually collected samples reflected the true prevalence of contaminated agents in the modeled operation. The zone 3 only (scenario 4) and model-based (scenario 5) sampling scenarios consistently overestimated true prevalence across time, suggesting that those scenarios could provide a more sensitive approach for determining if *Listeria* is present in the operation. The random sampling scenario (scenario 3) may be more useful for operations looking for a scheme that is most likely to reflect the true prevalence. Overall, the developed models allow for rapid virtual experimentation and evaluation of sampling schemes specific to unique fresh-cut produce facilities.

**IMPORTANCE** Programs such as environmental monitoring are used to determine the state of a given food facility with regard to the presence of environmental pathogens, such as Listeria monocytogenes, that could potentially cross-contaminate food product. However, the design of environmental monitoring programs is complex, and there are infinite ways to conduct the sampling that is required for these programs. Experimentally evaluating sampling schemes in a food facility is time-consuming, costly, and nearly impossible. Therefore, the food industry needs science-based tools to aid in developing and refining sampling plans that reduce the risk of harboring contamination. Two agent-based models of two fresh-cut produce facilities reported here demonstrate a novel way to evaluate how different sampling schemes can be rapidly evaluated across multiple time points as a way to understand how sampling can be optimized in an effort to locate the presence of *Listeria* in a food facility.

## INTRODUCTION

Listeria monocytogenes is a foodborne pathogen of high concern to the ready-to-eat food sector (1) as it can survive in and move through food facilities over time (2), potentially contaminating food products that do not undergo a kill step before consumption. Food facilities identify *Listeria* contamination routes or lapses in sanitation by using strategies such as environmental monitoring programs (EMPs) (3). EMPs involve the collection of sponge samples from the facility environment (e.g., equipment, floors, walls), which are tested for *Listeria* spp., with a positive test for *Listeria* spp. indicating that the pathogenic species L. monocytogenes is potentially, but not necessarily, present. Therefore, *Listeria* species testing is considered a more conservative approach for the identification of potential L. monocytogenes harborage points (4). The EMP samples are collected from sites that are typically categorized by “zone” (5). Although there are multiple ways for zone categorization, the zones discussed in this paper represent the following: zone 1, food contact surfaces such as conveyor belts; zone 2, surfaces in close proximity to food or food contact surfaces such as equipment frames; zone 3, sites within the production room but not in close proximity to food contact surfaces such as drains; and zone 4, sites outside of the production room such as break rooms. Given the complex nature of the food facility environment, including the complexity of each individual piece of equipment and the food product or people that move over or around it, there are significant challenges in identifying the best sampling sites for a sampling program as part of an EMP. There have been multiple recommendations for where and when to take samples, with recommendations on the number of samples to collect per zone and the number of hours after the start of production when samples should be collected (3–5). As it is impractical to sample the whole environment and nearly impossible to conduct an unbiased (e.g., controlled experiment) “trial and error” strategy of evaluating each of these sampling schemes, there is a need for science-based tools that can rapidly evaluate various sampling schemes at multiple time points.

Mathematical modeling has been explored over the last several years as an alternative approach to understanding *Listeria* in the environment, on foods, and in food retail and facility environments. For example, the U.S. Department of Agriculture (USDA) developed a quantitative risk assessment (QRA) model to mathematically simulate *Listeria* in a retail deli environment (i.e., “virtual deli”) (6). The USDA has also developed a risk assessment model that is a dynamic in-plant Monte Carlo model that predicts L. monocytogenes concentrations at retail (7). This model was used to evaluate the impact on public health of different scenarios of food contact surface sampling. A compartmental mathematical model was used to understand *Listeria* cross-contamination in a fish processing environment (8). More recently, agent-based models (ABMs) have been used as a way to model *Listeria* in food facilities, as these models have the advantage of being able to simulate the various interacting elements within these complex systems (9, 10). All of these models provide valuable insights into the risk of *Listeria* contamination of food or food contact surfaces. However, none investigate sampling scenarios associated with EMPs, such as location and timing of sample collection. Additionally, no models to date are specific to fresh produce facility environments. The produce industry needs more information on *Listeria* dynamics in produce operations to better prevent the occurrence of produce-associated outbreaks (11). There is a lack of science-based tools to implement responses to *Listeria* detection that are both appropriate for a specific facility and its unique processes and effective in reducing risk of contaminated products. The produce industry needs a way to save time and money in evaluating sampling scenarios by using science-driven processes for evaluating various sampling schemes instead of evaluating sampling schemes using trial and error. More specifically, there is a need for food facilities to rapidly evaluate how changes to inputs such as incoming product quality may change the optimal sampling scheme, across time or across operations. Ultimately, these models need to provide actionable and practical data to support feasible implementation of an optimal EMP. The objective of this study was to develop agent-based models specifically tailored to two fresh-cut produce facilities and use them to evaluate, *in silico*, multiple sampling scenarios. Specifically, the goal of such detailed modeling was to generate synthetic data via simulation of *Listeria* dynamics in the two fresh-cut produce facilities to determine if site-specific attributes impact the likelihood of contamination and therefore necessitate different sampling and surveillance schemes.

## RESULTS

**FIG 1:**
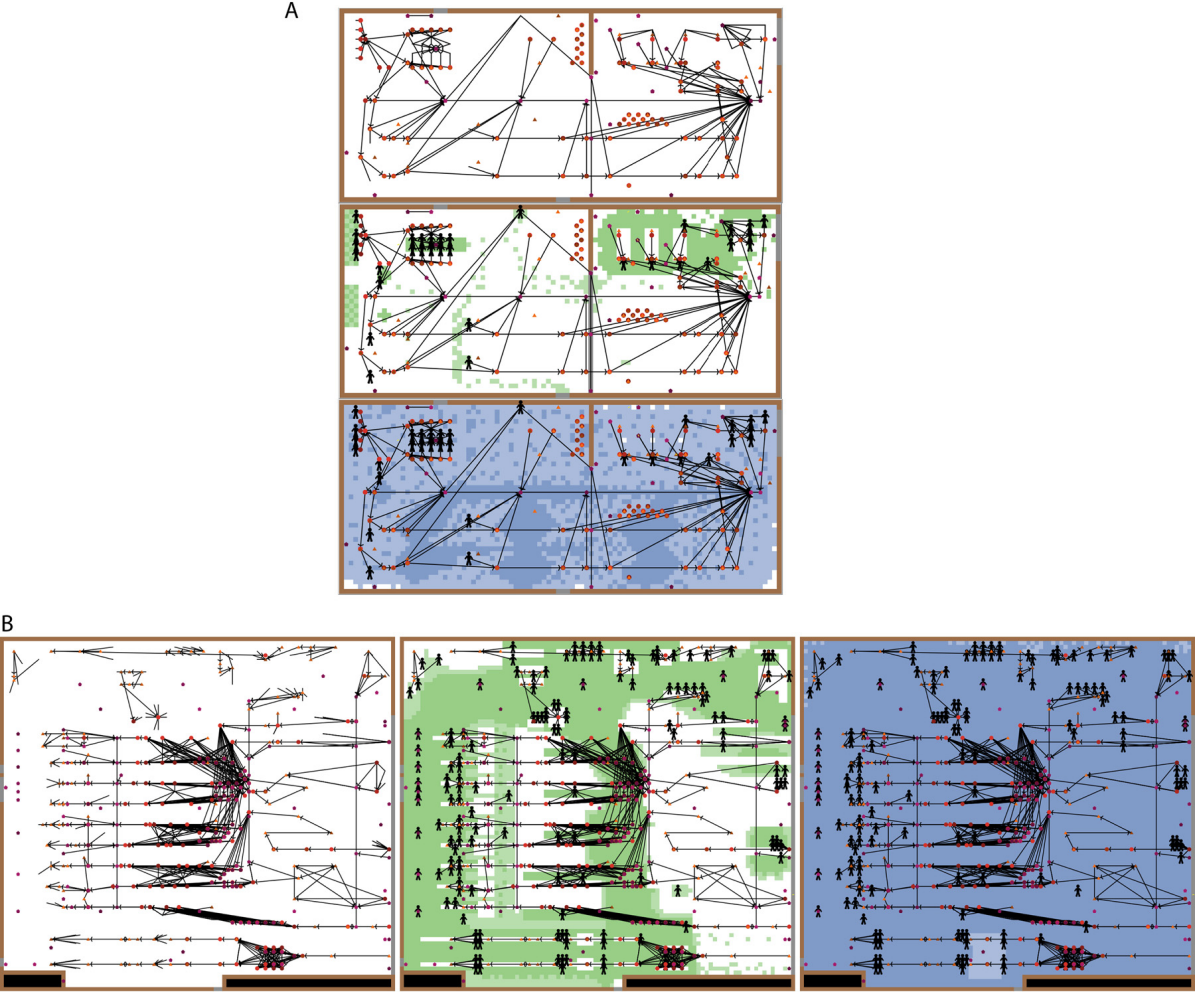
NetLogo model interface showing a 2D representation of the layout for facility A (A) and facility B (B). In both panels A and B, the first panel shows the locations of agents (symbols) and links connecting them (lines) in the modeled facility, while the second and third panels illustrate the traffic density (green) and water density (blue), respectively, on the floor at a particular point in time.

Agent-based models of two fresh-cut produce facilities were developed from code of a previously developed agent-based model, EnABLe (9) (Fig. 1; Table 1) with model parameters that were informed by (i) in-person observations, (ii) published literature, or (iii) an expert elicitation (Table 2; see Table S1 in the supplemental material). *Listeria* contamination routes and presence on surfaces during facility operations were simulated using “agents” representing equipment and employees, each having customized characteristics. The agents operated autonomously with other agents and the environment, including the floors, walls, and ceiling. Model validation was deemed successful based on overlap of the model’s predicted *Listeria* prevalence of contaminated agents (presented as percentiles, mean, median, and maximum) and observed prevalence in historical data (presented as the mean and 5% and 95% confidence intervals) (Tables 3 and 4). Sensitivity analysis was used to identify significant input parameters affecting prevalence in the overall model, while cluster analysis was used to determine the impact of agent attributes on contamination risk. The developed and validated models simulated the *Listeria* dynamics within the facilities of interest over a 2-week period and were used to evaluate the spread of *Listeria* and the sampling performance of different sampling scenarios.

**TABLE 1:** Summary of agent characteristics by zone and employees in agent-based models of two fresh-cut facilities

Facility and characteristic	Value^*a*^ for:			
	Zone 1^*b*^	Zone 2	Zone 3	Employees
Facility A				
No. of agents	130	120	30	34
Distance from floor (m)	1.2 (0.9, 4.6)	0.9 (0.9, 4.6)	0.9 (0.0, 1.8)	2.1 (1.2, 2.7)
Surface area (cm^2^)	7,432 (156, 66,147)	15,396 (914, 92,903)	3,871 (625, 15,396)	156 (156, 156)
No. of out-directed links	0 (0.0, 1.0)	0 (0.0, 1.0)	0 (0.0, 0.0)	0 (0.0, 0.0)
No. of in-directed links	0 (0.0, 1.0)	0 (0.0, 0.0)	0 (0.0, 6.6)	0 (0.0, 1.0)
No. of undirected links	1 (1.0, 4.0)	1 (0.0, 2.0)	1 (0.0, 5.2)	1 (1.0, 4.4)
No. (%) uncleanable	3 (2.3)	2 (1.7)	11 (36.7)	0 (0.0)
				
Facility B				
No. of agents	321	219	158	155
Distance from floor (m)	1.2 (0.3, 3.0)	1.2 (0.9, 4.0)	0.3 (0.0, 3.4)	1.2 (1.2, 1.5)
Surface area (cm^2^)	6,000 (156, 22,296)	15,000 (5,000, 33,445)	5,000 (729, 10,000)	156 (156, 156)
No. of out-directed links	0 (0.0, 2.0)	1 (0.0, 2.0)	0 (0.0, 1.0)	0 (0.0, 0.0)
No. of in-directed links	0 (0.0, 1.0)	0 (0.0, 1.0)	0 (0.0, 4.0)	0 (0.0, 0.0)
No. of undirected links	1 (1.0, 8.0)	1 (0.0, 3.0)	1 (0.0, 2.0)	1 (0.0, 8.0)
No. (%) uncleanable	34 (10.6)	34 (15.5)	41 (25.9)	0 (0.0)

a Values given are median (5th–95th percentile), unless otherwise stated.

b Agents representing employees were considered zone 1 and therefore are also included in the zone 1 agent summary.

**TABLE 2:** Input parameters for agent-based models of two fresh-cut produce facilities^*a*^

Symbol	Description	Equation/distribution	Mean	5th–95th percentile	Reference or source
*p_z_*	Probability that *Listeria* is introduced into the room via objects from zone 4 (e.g., trolley, cart, product bins)	10^PERT (−2.3, −0.9, −0.2, 4.8)^	0.14	0.02, 0.36	Expert opinion
*N_z_*	Amt of *Listeria* introduced per object from zone 4 (CFU)	10^PERT (0, 1.9, 3.3, 4.2)^	155.79	6.04, 618.79	Expert opinion
*R_d_*	Prevalence of *Listeria* in produce on day *d*, for *d* = Monday, Tuesday, Wednesday, Thursday, Friday	10^PERT (−2.3, −0.6, −0.6, 5.4)^	0.16	0.06, 0.24	Expert opinion
*N_R_*	Concn of *Listeria* per contaminated produce (CFU/g)	Gamma (0.0019, 0.019)	0.10	0.00, 0.00^*b*^	42
α	Proportion of *Listeria* transferred to an equipment surface upon contact with a contaminated produce	10^Normal (−0.28, 0.2)^	0.56	0.25, 1.00	43
*p_r_*	Rate of random event introducing *Listeria* (e.g., drain backs up, maintenance, roof leak) (h^−1^)	(1/10)^PERT (−4.3, −0.9, −0.6, 4.6)^	0.07	0.00, 0.20	Expert opinion
*p_s_*	Probability of random introduction to ceiling		0.05		Assumed
	Probability of random introduction to floor		0.85		Assumed
	Probability of random introduction to equipment		0.10		Assumed
*N_r_*	Amt of *Listeria* introduced per random event (CFU)	10^PERT (0.2, 3.3, 3.7, 3.3)^	1,233.63	41.56, 3,828.76	Expert opinion
*K*	Environmental carrying capacity of *Listeria* (CFU/ml)		10^5^		44
GT	Generation time (h) of *Listeria* on environment surfaces (5–8°C)	Uniform (47, 155)	101.00	52.40, 149.60	33
μ	Maximum specific growth rate (h^−1^) of *Listeria* on environment surfaces (5–8°C)	=ln(2)/GT	0.01	0.00, 0.01	Calculated
*p_t_*	Probability that contact on floor from foot and equipment traffic is sufficient to spread *Listeria* to adjacent patch	PERT (0.03, 0.25, 0.65, 4)	0.28	0.10, 0.48	45
*c_i_*	Contact rate between the contaminated patch and the adjacent patch given the traffic level *i* = high, low, negligible	*c*_high_ = 60/patch/h, *c*_low_ = 12/patch/h, *c*_neg_ = 0.2/patch/h			Observed
*p_w_*	Probability that environmental *Listeria* is transported to adjacent patches via (visible) water	Uniform (0.01, 0.05)	0.03	0.01, 0.05	Assumed
β	Transfer coefficient for *Listeria* transmission among patches via traffic and water	Uniform (0.0, 0.05)	0.03	0.00, 0.05	Assumed
*p_f_*	Probability that produce falls to the floor during any given hour of production				
	Facility A	Uniform (0.05, 0.10)	0.08	0.05, 0.10	Observed
	Facility B	Uniform (0.00, 0.01)	0.00	0.00, 0.01	Observed
*p_c_*	Probability of a condensation transfer event given *Listeria* is present	Uniform (0.01, 0.02)	0.02	0.01, 0.02	Assumed
*η_d_*	Log10 reduction of *Listeria* from washing and sanitation on day *d*, for *d* = Monday, Tuesday, Wednesday, Thursday, Friday	PERT (−8, −6, −1.5, 4)	−5.58	−7.36, −3.47	44
γ	Probability that a cleanable agent was not properly cleaned at the end of the shift	Uniform (0.95, 1.00)	0.98	0.95, 1.00	Assumed
τ*ij*	Probability of *Listeria* species transfer from agent *i* to *j* given contact, where *i *=* j* = zone 1, zone 2, zone 3, or employee agent type^*c*^	10^normal (tc, STD)^			43, 46

a Parameters were identical between the two facility models, with the exception of *p_f_*, which represents the probability that food will fall to the floor. All parameter values correspond to an hourly time scale, the time scale of the model.

b This is a highly skewed distribution, as evidenced by a 99th percentile of 0.14 CFU/g and a maximum of 380.82 CFU/g.

c tc, mean transfer coefficient; STD, standard deviation of the transfer coefficient. Complete data are given in Tables S2 and S3 in the supplemental material.

**TABLE 3:** Model output versus historical data for validation of *Listeria* prevalence in an agent-based model of fresh-cut produce for facility A^*a*^

Equipment category	Model probability of contamination						Historical data				
	Mean^*b*^	Median	5th percentile	95th percentile	99th percentile	Max	No. tested	No. positive	Probability of contamination	5% CI^*c*^	95% CI^*c*^
Control panel	0.001	0.000	0.000	0.000	0.000	1.000	9	0	0.00	0.00	0.30
Door	0.016	0.000	0.000	0.000	1.000	1.000	10	0	0.00	0.00	0.28
Drain	0.099	0.000	0.000	0.667	1.000	1.000	11	0	0.00	0.00	0.26
Floors	0.004	0.000	0.000	0.000	0.143	0.500	65	0	0.00	0.00	0.06
Frame	0.029	0.000	0.000	0.200	0.333	1.000	39	0	0.00	0.00	0.09
Ladder	0.003	0.000	0.000	0.000	0.000	1.000	10	0	0.00	0.00	0.28
Miscellaneous	0.001	0.000	0.000	0.000	0.000	1.000	15	0	0.00	0.00	0.20
Packing	0.016	0.000	0.000	0.000	1.000	1.000	4	0	0.00	0.00	0.49
Squeegee	0.000	0.000	0.000	0.000	0.000	0.000	12	0	0.00	0.00	0.24
Trash (gray bins)	0.001	0.000	0.000	0.000	0.000	1.000	5	0	0.00	0.00	0.43
Trash (white bins)	0.000	0.000	0.000	0.000	0.000	1.000	4	0	0.00	0.00	0.49
Trash (yellow bins)	0.000	0.000	0.000	0.000	0.000	1.000	5	0	0.00	0.00	0.43
Wall	0.000	0.000	0.000	0.000	0.000	1.000	12	1	0.08	0.01	0.35
Weigher	0.002	0.000	0.000	0.000	0.000	1.000	5	0	0.00	0.00	0.43
Total	0.018	0.000	0.000	0.080	0.125	0.316	206	1	0.00	0.00	0.03

a *Listeria* prevalence is defined as the number of agents within a category that were positive for *Listeria* relative to the total number of agents within that category.

b Probability of contamination predicted by the model represents the average prevalence for all iterations.

c Five percent and 95% confidence intervals (CI) for historical data were calculated using a Wilson score interval, a binomial proportion confidence interval.

**TABLE 4:** Model output versus historical data for validation of *Listeria* prevalence in an agent-based model of fresh-cut produce for facility B^*a*^

Equipment category	Model probability of contamination						Historical data				
	Mean^*b*^	Median	5th percentile	95th percentile	99th percentile	Maximum	No. tested	No. positive	Probability of contamination	5% CI^*c*^	95% CI^*c*^
Chain	0.000	0.000	0.000	0.000	0.000	1.000	1	0	0.00	0.00	0.79
Door	0.063	0.000	0.000	1.000	1.000	1.000	13	1	0.08	0.01	0.33
Drain	0.175	0.000	0.000	1.000	1.000	1.000	18	2	0.11	0.03	0.33
Dryer	0.007	0.000	0.000	0.000	0.253	1.000	17	1	0.06	0.01	0.27
Floor	0.002	0.000	0.000	0.000	0.000	1.000	17	0	0.00	0.00	0.18
Frame	0.035	0.000	0.000	0.333	1.000	1.000	19	0	0.00	0.00	0.17
Mezzanine	0.001	0.000	0.000	0.000	0.000	1.000	2	0	0.00	0.00	0.66
Pallet jack	0.064	0.000	0.000	1.000	1.000	1.000	7	0	0.00	0.00	0.35
Table	0.000	0.000	0.000	0.000	0.000	0.000	1	0	0.00	0.00	0.79
Trash	0.286	0.000	0.000	1.000	1.000	1.000	1	0	0.00	0.00	0.79
Total	0.108	0.125	0.000	0.333	0.444	0.714	96	4	0.04	0.02	0.10

a *Listeria* prevalence is defined as the number of agents within a category that were positive for *Listeria* relative to the total number of agents within that category.

b Probability of contamination predicted by the model represents the average prevalence for all iterations.

c Five percent and 95% confidence intervals (CI) for historical data were calculated using a Wilson score interval, a binomial proportion confidence interval.

### The baseline model predictions show that agents in zone 3 and the second production shift had the greatest *Listeria* prevalence.

**FIG 2:**
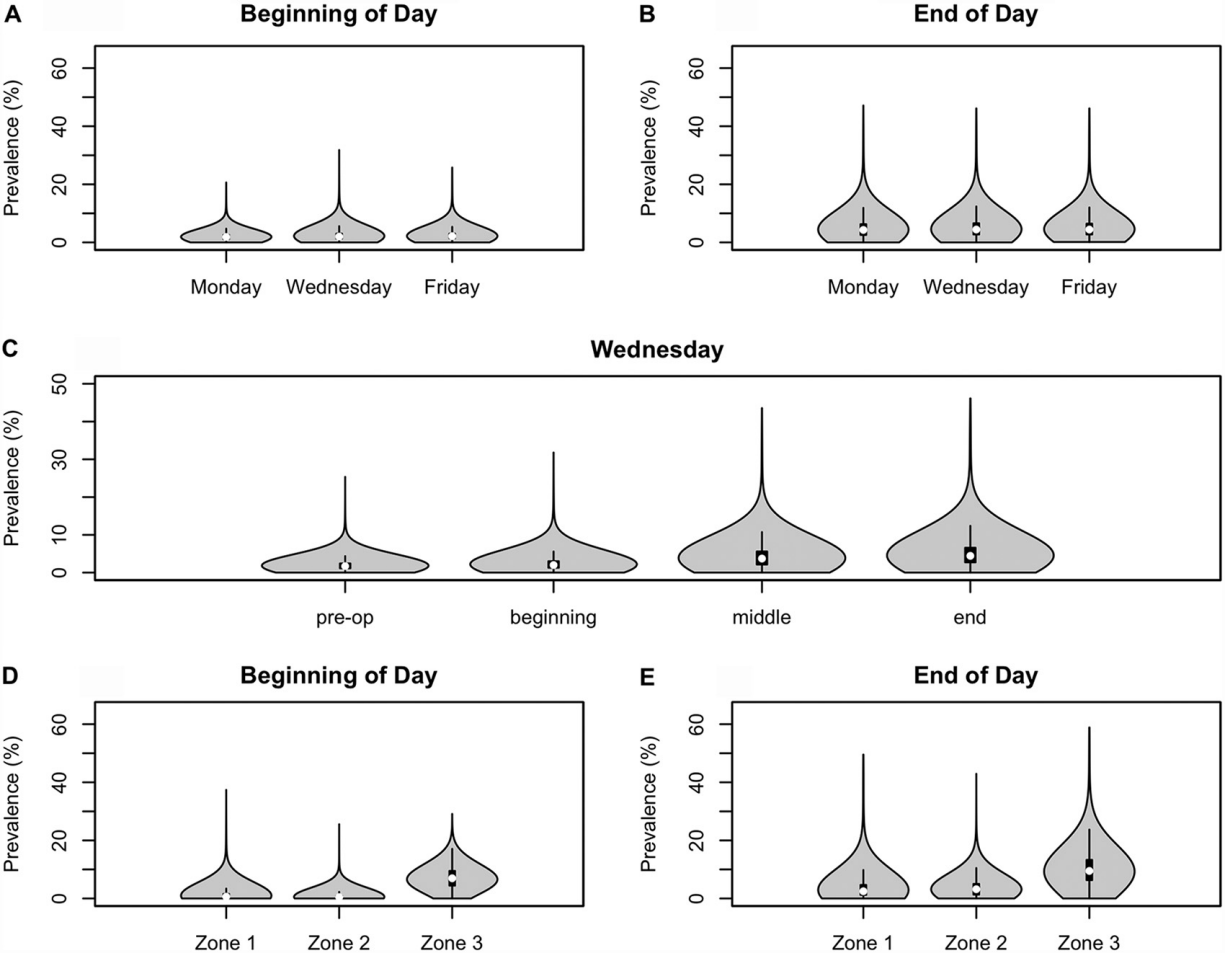
Simulation analyses of the baseline model for facility B. Violin plots show the distribution of the *Listeria* prevalence from 10,000 iterations, with a white circle representing the median and a black rectangle representing the interquartile range. The model outcomes were compared by day of the week at the beginning of the day (A), day of the week at the end of the day (B), time of day throughout Wednesday (C), zone at the beginning of the day (D), and zone at the end of the day (E) (employees are considered zone 1).

*Listeria* prevalence among modeled agents (i.e., the proportion of agents contaminated with *Listeria* out of all modeled agents) during the second week of a 2-week simulation for the baseline models for both facilities was evaluated based on the results of 10,000 iterations. The overall *Listeria* prevalence in a given day remained similar throughout the week (Fig. 2A and B) (for brevity, data are shown only for facility B). However, *Listeria* prevalence was, on average, greater at the end of the day (18th hour of production) than at the beginning of the day (first hour of production) (Fig. 2C), regardless of the day of the week. Agents from zone 3 had a higher *Listeria* prevalence, on average, than agents in zones 1 and 2 (Fig. 2D and E). This was true for both the start of the day and the end of the day. Similar trends were observed for both facilities.

### Sensitivity analysis revealed key parameters that consistently influenced *Listeria* prevalence in all zones within the facilities modeled here.

**FIG 3:**
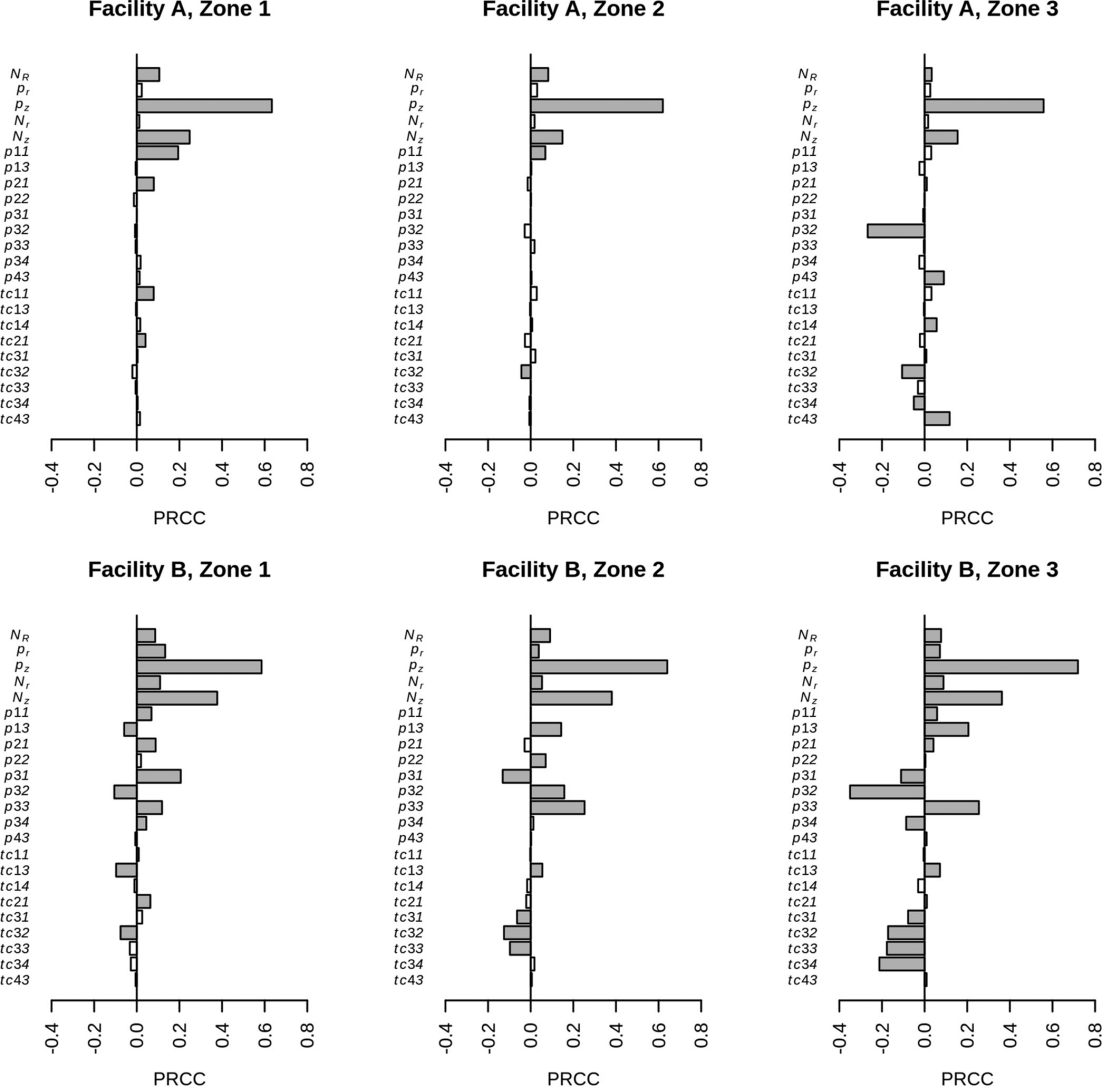
Sensitivity analysis based on partial rank correlation for facility A (top) and facility B (bottom) reveals key parameters significantly affecting *Listeria* presence on agents from zones 1, 2, and 3 (employees are considered zone 1) at the end of the shift on Wednesday in week 2. Gray bars indicate significant parameters (*P* < 0.05/53 after Bonferroni’s correction), while white bars indicate nonsignificant parameters. *N_R_*, concentration of *Listeria* spp. per contaminated produce (CFU/g); *p_r_*, rate of a random event introducing *Listeria* spp.; *p_z_*, probability that *Listeria* spp. is introduced into the room via objects from zone 4; *N_r_*, amount of *Listeria* spp. introduced per random event (CFU); *N_z_*, amount of *Listeria* spp. introduced per object from zone 4 (CFU); *pij*, probability of contact from contaminated zone *i* to zone *j*; *tcij*, probability of *Listeria* spp. transfer from zone *i* to zone *j* given contact (parameter notations are defined in Table 2 and Table S1).

Sensitivity analyses based on partial rank correlation were performed within each modeled facility to investigate which parameters had the greatest influence on *Listeria* prevalence by zone (i.e., number of agents of a given zone that were positive for *Listeria* relative to the number of total agents within that zone) (Fig. 3). The analysis revealed that three input parameters were significant for both facilities in all zones: (i) the probability of zone 4 introduction (i.e., input parameter *pz*), (ii) the amount of *Listeria* introduced per object from zone 4 (*N_z_*), and (iii) the concentration of *Listeria* per contaminated incoming produce (*N_R_*). The rate of introduction from random events (*pr*) and the amount of *Listeria* introduced during random events (*N_r_*) were significant for all zones in facility B but were not influential on equipment contamination in any zone in facility A. The probability of *Listeria* transfer from zone 2 to zone 1, given contact, had a significant influence on zone 1 contamination in both modeled facilities.

### Cluster analysis provided an alternative to grouping sites by zone and revealed that groupings differ across the two facilities.

A hierarchical cluster analysis was performed to determine how agents across both facilities (i.e., equipment and employees) grouped with respect to predicted *Listeria* contamination risk outcomes (e.g., frequency, probability, and duration of contamination) and to determine whether there were patterns within and across facilities. The results revealed an alternative method for categorizing sampling sites (Table 5), as opposed to grouping by zones based on proximity to food. Ultimately, three clusters were identified that were each comprised of agents from all three zones. Cluster I was the largest of the three clusters. Compared to clusters II and III, agents in cluster I had, on average, the fewest contacts by a contaminated agent, the lowest time contaminated over the course of a 2-week simulation, and the least amount of consecutive time contaminated (i.e., 11 h). Cluster II was the second largest cluster. Nearly all agents within this cluster (97%) were considered cleanable, with all of the uncleanable agents in this cluster being from facility B. Agents in this cluster were the greatest distance from the floor, compared to the other two clusters. For cluster II, agents were consecutively contaminated, on average, for 26 h, suggesting that they remain contaminated after cleaning. Cluster III was the smallest cluster. The agents within this cluster from zones 1 and 2 were from facility A, while all of the zone 3 agents were from facility B. All of the cleanable agents from cluster III were from facility A, while all of the uncleanable agents were from facility B. The facility A agents within cluster III represented packing machines. The facility B agents within cluster III represented drains, pallet jacks, and trash equipment. Overall, the majority of the agents in cluster III (88%) were uncleanable. The agents in cluster III had the greatest average time contaminated and the greatest average consecutive time contaminated (i.e., 259 h). Cluster III agents also had, on average, the greatest number of contacts by contaminated agents, the greatest number of transfers of contamination, and the greatest number of times an uncleanable site becomes contaminated (Table 5). Overall, all three clusters comprised agents from all three zones, thus providing an alternative way to categorize agents beyond the traditional zone categorization that is based on an agent’s proximity to food.

**TABLE 5:** Groups of agents in agent-based models of fresh-cut facilities A and B identified by cluster analysis based on several agent *Listeria* contamination risk outcomes^*a*^

Agent parameter	Value for agent groupings (*n* = 978) by cluster		
	I	II	III
Total no. of agents	858	103	17
No. of agents by facility
A	236	42	2
B	622	61	15
No. of agents by zone
1	379	71	1
2	317	21	1
3	162	11	15
Representative agent(s)			
No. with cleanability
Yes	751	100	2
No	107	3	15
Distance from floor (m)^*b*^	4.33	5.10	1.00
No. of out-directed links^*b*^	0.50	0.71	0.06
No. of in-directed links^*b*^	0.51	0.41	1.71
No. of undirected links^*b*^	1.82	1.47	1.59
Probability of *Listeria* contamination^*b*^	0.01	0.05	0.67
Contacts by contaminated agent (per 2 wks, via link)^*b*^	0.93	16.57	68.65
Transfers of contamination (per 2 wks, via link)^*b*^	0.93	22.37	42.53
Time contaminated (h)^*b*^	0.74	30.92	195.30
Maximum consecutive time contaminated (h)^*b*^	11.34	26.10	258.80
No. of times an uncleanable site becomes contaminated (per 2-wk simulation)^*b*^	0.03	0.12	6.68
No. of times agents were contaminated from incoming food product (per wk)^*b*^	0.02	1.41	0.00
No. of times agents were contaminated from objects coming into the room (i.e., from zone 4) (per wk)^*b*^	0.01	1.31	2.47

a Agent *Listeria* contamination risk outcomes included, e.g., probability, frequency, and duration of contamination.

b Mean of cluster.

### Sampling performance differed throughout production and as true prevalence increased.

**FIG 4:**
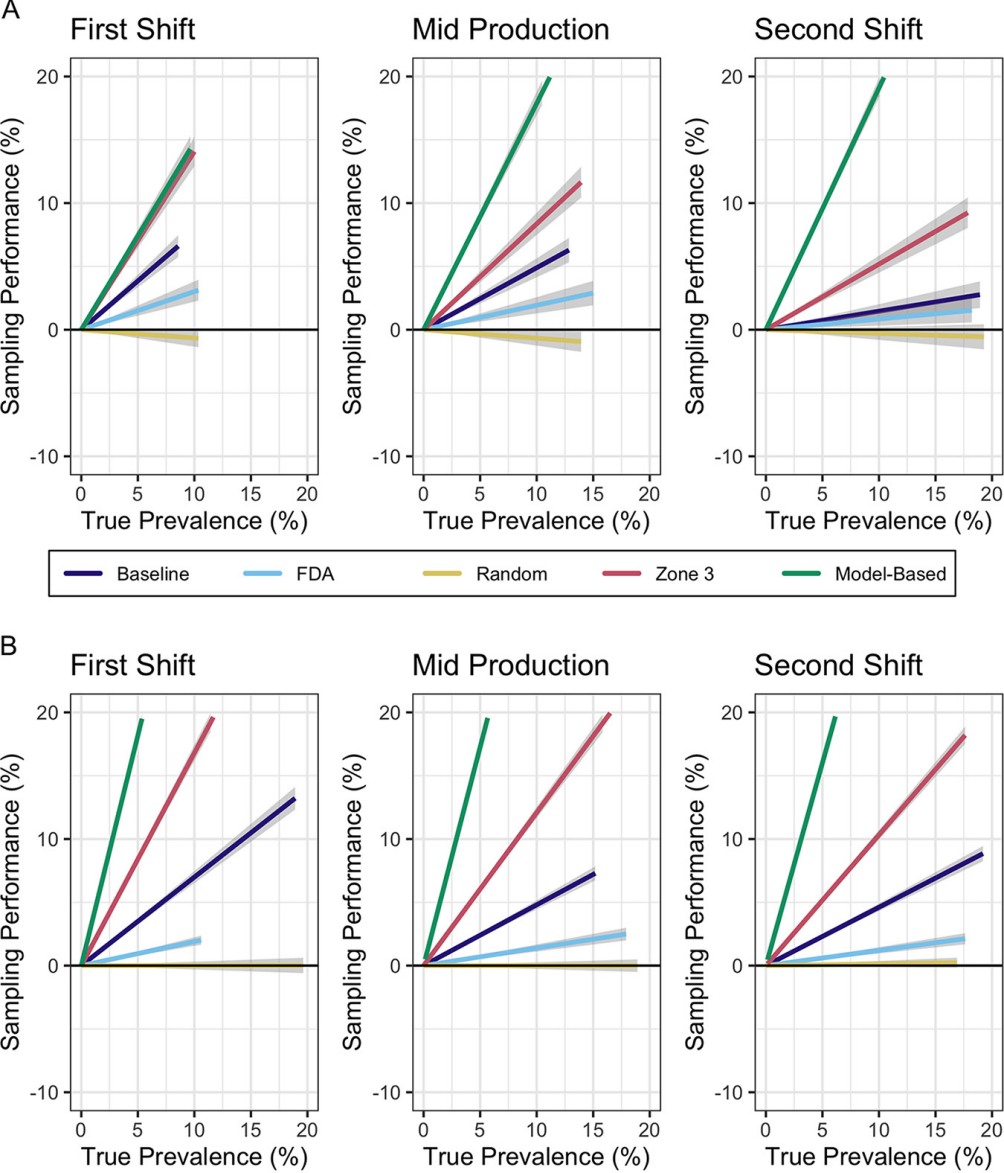
Sample performance relative to true prevalence for five sampling scenarios at three time points on Wednesday for agent-based models of fresh-cut produce facilities A (A) and B (B). Sampling scenarios are the following: Baseline, facility’s current environmental monitoring program; FDA, FDA recommendations; Random, random selection; Zone 3, sites exclusively from zone 3, which are sites in the production room but not directly adjacent to food contact surfaces; and Model-Based, model prediction of elevated risk of contamination. Sampling performance was calculated as the proportion of contaminated agents among all samples minus true prevalence (i.e., the proportion of contaminated agents among all agents). The line of best fit for each scenario represents the average sampling performance across 1,000 iterations per scenario-time combination compared to the true prevalence at that time. A sampling scenario that had a *Listeria* prevalence representative of the true prevalence would align with *y* = 0. Shaded regions represent 95% confidence intervals.

Scenario analyses determined how sampling approaches differed based on five proposed sampling schemes conducted at three different times in a day, resulting in 15 scheme-time combinations (i.e., scenarios, as modeled using scenario analysis) (Table 6). Information provided by each facility about their in-house sampling plan was used to determine the total number of samples collected for the sample scenarios. The “diagnostic” accuracy of each sampling scenario was measured by the sampling performance versus the “true prevalence” (i.e., the number of agents contaminated at a point in time with *Listeria* out of the total number of agents in the model). Through this approach, we used the developed models to infer, for the real facilities, how successful different sampling scenarios are at capturing the true contamination prevalence (across all possible environmental surfaces) in the real facility, which would be nearly impossible to determine. For example, in a plot of sampling performance versus true model prevalence, a sampling scenario with *Listeria* prevalence equal to the true prevalence would align with *y* = 0. A sampling scenario that falls below this line would indicate that the sampling scenario underestimates the *Listeria* prevalence of the operation. If the goal of the sampling plan was to find *Listeria* if it is present, then a positive sloping line (overestimation of true prevalence on average) would indicate a sampling scheme that is successful. Scenario analysis results were graphically compared (Fig. 4A and B) using a line of best fit and the corresponding slope (Table 7) to evaluate the five different sampling scenarios against true prevalence. For facilities A and B, scenario 1, which represented the facility’s existing sampling plan, had a positive slope for all three time points: the first shift (first hour of production), midproduction (hours 3 to 4 of production), and the second shift (hours 9 to 10 of production). Scenario 2, which represented the draft FDA recommendation, also had a positive slope for all time points in both facilities but had a slope close to zero for the second shift time point. Scenario 3, representing samples collected “randomly” (i.e., all agents had an equal chance of being sampled), had approximately zero slope for all time points in facility B and a negative slope for all time points in facility A (although all slopes were close to zero [i.e., between 0 and −0.1]). Scenario 4 represented samples collected from only zone 3 agents, which was the zone with the fewest agents. Scenario 4 resulted in a consistently greater slope across all time points than the previous three scenarios. A positive slope for the scenario that used only zone 3 agents was not unexpected, as *Listeria* prevalence by zone (Fig. 2D and E) showed that zone 3, in addition to being the zone with the fewest number of agents, had the greatest prevalence on average. Scenario 5, representing sample selection based on the cluster analysis grouping of model prediction, had a consistently greater slope across all time points than that of the previous four scenarios.

**TABLE 6:** Number of samples collected from each zone for each of four simulated sampling scenarios (1 to 4) and the number of samples collected from each cluster as determined by the model in an additional simulated sampling scenario (5)^*a*^

Model and zone	No. of samples for indicated sampling scenario				5. Model-based^*f*^	
	1. Baseline^*b*^	2. FDA^*c*^	3. Random^*d*^	4. Only zone 3^*e*^	Cluster	No. of samples
Model A						
Zone 1	0	10	30 (random)	0	I	8
Zone 2	10	10		0	II	20
Zone 3	20	10		30	III	2
						
Model B						
Zone 1	0	35	105 (random)	0	I	29
Zone 2	45	35		0	II	61
Zone 3	60	35		105	III	15

a All five scenarios were evaluated by the agent-based models for fresh-cut produce facilities A and B through testing them at three time points: first hour of production, fourth hour of production, and tenth hour of production.

b The numbers of samples for the baseline scenario were adapted from the facility’s current sampling plans.

c The numbers of samples for the FDA scenario were based on the recommendation provided by the FDA draft guidance (41), which states “We recommend that even the smallest processors collect samples from at least 5 sites of FCS and 5 sites of non-FCS on each production line for RTE foods.” Facility A had two lines. Facility B had seven lines. FCS, food contact surface; RTE, ready to eat.

d The numbers of samples collected for the random scenario were randomly selected from any of the three zones.

e The numbers of samples for the “only zone 3” scenario included only samples from zone 3 (i.e., sites in exposed product room but not directly adjacent to food contact surfaces, such as drains).

f The model-based scenario was based on cluster analysis, with the majority of sites coming from agents in clusters II and III due to those clusters having greater *Listeria* contamination, on average. The number of samples collected from cluster III (and cluster II in the case of model B) was limited by the number of agents that were categorized into that cluster.

**TABLE 7:** Slope for linear regressions fitted to the predicted sampling performance for different true prevalences in the facility in 1,000 iterations of each scenario-time combination for agent-based models of two fresh-cut produce facilities

Time^*a*^	Scenario^*b*^^,^^*c*^^,^^*d*^^,^^*e*^^,^^*f*^	Slope	
		Facility A	Facility B
First shift	1. Baseline	0.770	0.667
	2. FDA	0.301	0.178
	3. Random	−0.065	0.008
	4. Only zone 3	1.407	1.581
	5. Model based	1.480	3.365
Midproduction	1. Baseline	0.430	0.386
	2. FDA	0.193	0.121
	3. Random	−0.067	0.020
	4. Only zone 3	0.747	1.068
	5. Model based	1.841	3.203
Second shift	1. Baseline	0.123	0.380
	2. FDA	0.083	0.101
	3. Random	−0.046	0.023
	4. Only zone 3	0.374	0.939
	5. Model based	1.935	2.901

a All five scenarios were tested at three time points: 1 h into production, 4 h into production, and 10 h into production.

b The numbers of samples for the baseline scenario were adapted from the facility’s current sampling plans.

c The numbers of samples for the FDA scenario were based on the recommendation provided by the FDA draft guidance (41), which states “We recommend that even the smallest processors collect samples from at least 5 sites of FCS and 5 sites of non-FCS on each production line for RTE foods.” Facility A had two lines. Facility B had seven lines. FCS, food contact surface; RTE, ready to eat.

d The numbers of samples collected for the random scenario are randomly selected from any of the three zones.

e The numbers of samples for the “only zone 3” scenario included only samples from zone 3 (i.e., sites in exposed product room but not directly adjacent to food contact surfaces, such as drains).

f The model-based scenario was based on cluster analysis, with the majority of sites coming from agents in clusters II and III due to those clusters having greater *Listeria* contamination, on average.

## DISCUSSION

Two agent-based models were created to simulate *Listeria* dynamics in fresh-cut produce facilities. Both models had a user interface with the facility layout, the location of agents (e.g., equipment, employees), zone designations for nonemployee agents, connections between agents, and viewing controls for water and traffic maps (Fig. 1). In addition to visualization of contamination risks and pathways, analyses of the models produced detailed simulation-based site-specific data on frequency, timing, level, and transmission of *Listeria* contamination in each facility (Fig. 2). The customized models allowed for rapid virtual experimentation and evaluation of a variety of EMP sampling scenarios.

### *Listeria* dynamics in fresh-cut produce facilities can be modeled effectively using an *in silico* approach.

The model was developed for use in fresh-cut produce operations to the best of our ability given the available data. Given that there are several challenges to understanding and predicting *Listeria* contamination in a food facility, it is important to understand that our model, as with any model, is a simplification of reality. It is particularly challenging to model food facilities with low historical prevalence detected and low sample numbers, as it is difficult to validate the absence of evidence of contamination or to predict “rare events” (12), as is sometimes the case with *Listeria* contamination. Compared to previous studies of similar models where prevalence was higher (9, 10), this challenge was unique to the facilities modeled in our study. The validation done here used robust variable selection, an approach similar to a previous rare-event study (13). Future further validation of the model with facility-specific data would be valuable, but more stringent validation than reported here would likely rely on confidential data and would thus occur by end users. Thus, even with the limited validation data, the models developed here represent an important framework and next step in facilitating further development and use of ABMs in food safety. While future efforts should consider more extensive sample collection to obtain validation data, we acknowledge that validation of models in real processing facilities can be challenging due to the dynamic nature of processing operations, including the need for continuous improvement of operations to address issues that are revealed by both data collection and modeling efforts. Despite these limitations, ABMs represent decision support tools that will still need to be used in conjunction with robust real data in decision-making. Overall, these models illustrate the real-life difficulties in designing EMPs when the sampling effort is low due to cost and logistic constraints or if the sampling effort may not be representative of reality (e.g., in cases where the sampling strategy intentionally or unintentionally finds no positives in a facility over a substantial time frame). For example, for categories where fewer historical data were available (i.e., only a few samples were collected from sites in a given category), the 95% confidence intervals were wide, demonstrating a challenge of attempting to validate a model in parts rather than as a whole, as fewer samples means a higher level of uncertainty described with wider confidence intervals. Importantly, the predicted prevalence for both models was on the lower end of the confidence intervals of the historical data, suggesting a potential limitation, as the models may, in general, be underestimating *Listeria* prevalence. This indicates that *Listeria* may be introduced, transferred, or harbored in a way that the models do not currently consider. For example, one limitation of the model was that the product flow was based on that of a typical day and the model did not account for seasonal variations that may result in increased product flow.

Additionally, validation is difficult for a model that represents a real-life facility, as a facility can change over the time (e.g., addition or removal of equipment) for which historical sampling data are available. For example, facility A underwent construction midway through historical data collection, and there may be other events or changes that were not known to the modelers and may even be unknown to the facility managers. Therefore, the model that was developed is reflective of the state of the facility that existed during the second half of historical data collection, but the validation data were from both before and after that construction (using data from after the reconstruction only would have yielded a prohibitively small validation data set). This is a recurrent limitation of the models, where in-person observations made regarding the location of employees and equipment and the relationships between them are not consistent week to week or even day to day. It is impossible to know how these changes affect *Listeria* prevalence. Models from previous studies generally used a theoretical food facility for the model, rather that modeling a specific operation, which often requires even further assumptions and simplifications (7, 10). Agent-based modeling has been done on a specified facility previously (9) and therefore had assumptions similar to those of the models discussed here. As is the case with all models, the models developed for our study have underlying assumptions and limitations. However, this does not prevent the use of the models to understand *Listeria* dynamics in food facilities and to facilitate discussion on the evaluation of optimal or risk-based sampling schemes. Despite these limitations, the developed models are still a valuable resource to the food industry as a tool for rapid experimentation to inform discussions related to development and refinement of sampling plans.

### Characteristics such as cleanability, connectivity, and distance from the floor should be considered during sample site selection.

A hierarchical cluster analysis was performed to evaluate how agents may be grouped together based on multiple predicted *Listeria* contamination risk outcomes. Three clusters grouped agents from both facilities and all zones. Interestingly, this suggests that learnings may be applicable to other facilities, as agents from the same facility did not appear to form their own clusters and had similar distributions of agents across clusters. Additionally, since agents from all zones were found in all clusters, zones may not be the ideal way to categorize sites when building a sampling plan. Instead, our study found that cluster III was dominated by agents described as uncleanable, suggesting that cleanability may be a driving factor in clustering rather than zone. Additionally, the agents within cluster III were, on average, at waist height and also had, on average, the greatest number of in-directed links (i.e., relationships with other agents that would transfer contamination, such as a trash bin that receives waste off a conveyor belt or a packing machine that receives product at the end of the line). It therefore may be valuable for an operation to create a site list of uncleanable sites, at waist height, and those with multiple in-directed links as a way to routinely sample high-risk sites. The food industry and scientific community have recognized that the hygienic design of equipment is a crucial part of preventing harborage of *Listeria* (14). The cluster analysis also revealed that agents that spent the greatest time contaminated were the agents that were most frequently a source of contamination for other agents. This suggests that agents that are considered uncleanable should be carefully reviewed for their relationships and proximity with other agents in the facility, when considering risk. Overall, these findings are consistent with clustering observed with the model developed by Zoellner et al. (9), who found that zones were not a good predictor of clusters. Although the use of zones is common in the food industry, alternative groupings similar to the clustering discussed above can be used to elucidate sampling results, such as was done in a study by Estrada et al., who used six categories including “drain sites” and “mobile nonfood contact surfaces” (15). Zone categorization provides information on the likelihood of contacting food products but does not provide any information on characteristics that may affect the risk of the site testing positive or becoming a niche (i.e., a site where *Listeria* survives over time), such as those characteristics identified in our study (e.g., cleanability). Therefore, it is important to consider alternative methods of systematically categorizing the sites to assist in sample site selection. For example, the facilities modeled here could create a list of sites that are more frequently sampled based on the agents identified here as being uncleanable and that have a proportionally high number of contacts toward the agent. The facilities could also review the list and determine ways to minimize the contacts that occur with these agents, such as restricting product or traffic flow patterns, or determine a way to make an uncleanable agent into a cleanable agent, such as by disassembly for cleaning.

### The developed models allow for rapid virtual experimentation and evaluation of a variety of sampling schemes.

Although sampling schemes have been evaluated in other industries (16, 17), and to a certain extent in the food industry for field testing (18), finished product testing (19, 20), and food contact surface testing (7), an evaluation of sampling schemes for environmental monitoring of food facilities has, to our best knowledge, not been previously published. The success of different sampling schemes currently utilized in the food industry was evaluated by comparing the sampling performance to the true prevalence from simulated production in the modeled facilities. The sampling performance for the majority of scheme-time combinations indicated overestimation of the overall contamination level of the facility. Depending on the definition of a successful sampling plan, this could be valuable. For example, if the goal of the sampling plan is to detect *Listeria* control issues as quickly and as soon as possible, this overestimation of true prevalence can provide a more sensitive approach for determining if *Listeria* is present in the operation. Previous studies have shown that sampling done by an outside expert to validate routine EMP sampling schemes can reveal significant differences in *Listeria* prevalence (21, 22). The overestimation of *Listeria* prevalence (as demonstrated by sample performance) was more pronounced at higher values of true prevalence, highlighting the potential for differences in risk perception among the scenarios’ sample allocation, particularly demonstrating scenarios 4 and 5 as more targeted approaches. Scenario 4 represents sites only from zone 3, and this finding is therefore consistent with the model, which found that *Listeria* prevalence was highest in zone 3, compared to zones 1 and 2. This makes sense, as zone 3 sites tend to include drains and other sites that have in-directed links from multiple sites and historically are known to capture the overall hygienic status of the room. Multiple studies have identified zone 3 sites such as drains as positive sites during *Listeria* environmental sampling (15, 21, 23, 24). Similarly, scenario 5 represented sites selected based on the cluster analysis of model predictions and thus targeted sites with characteristics conducive to becoming and remaining contaminated. Therefore, using a sampling scheme that targets sites based on identified problematic characteristics (which can be identified on new pieces of equipment that are brought into the operation) may be of greatest value for understanding where *Listeria* may be in a food facility.

Some would argue that a successful sampling plan is one that is closest to representing true prevalence (or true risk), even as true prevalence increases. Whereas the previous definition of success aimed at finding *Listeria* if and when present, this approach aims to provide an estimate of the extent of contamination throughout a facility. True prevalence is difficult to quantify, especially when the facility environment is not easily divided into sampling units, and when true prevalence is unknown, it is difficult to determine appropriate sampling levels for estimating risk. In other words, it is difficult to answer the common question “How many samples should I take in my facility?” In our evaluation of sampling scenarios, those closely representing true risk were those resulting in a line with a slope close to 0. These types of scenarios appear to perform uniformly across the range of contamination events that may occur in the food facility over time, thus providing a snapshot of the overall contamination status of the operation. This was the trend for the FDA-recommended scheme (scenario 2) and the random sampling approach (scenario 3) in both modeled facilities. The main benefit of these scenarios is that true risk was roughly matched by sampling only a subset of the environment, in fact the current sample numbers the facilities are using (30 in facility A and 105 in facility B), which is important when considering the time and resource costs of implementing routine EMPs. Future scenarios could evaluate how sample number impacts sampling performance.

Overall, these findings suggest that if facility personnel want to find *Listeria* if it is present in the operation, then scenario 4 would be most effective, as it more often identifies contamination among a fixed number of samples (i.e., overestimates the true prevalence). However, scenario 4 only collects samples from zone 3 (e.g., drains, equipment wheels, door frames, pallet jacks, trash bins, and conveyors, squeegees, ladders) and therefore does not provide any information about zones 1 and 2, which inherently have a higher risk of contaminating food. Scenario 2 or 3 would be preferred for facility personnel that want to have information about the overall operation, therein gaining information about the risk of *Listeria* contaminating product as well as an idea of the true prevalence. Interestingly, scenario 1, representing the facilities’ current sampling plans, overestimated the true prevalence compared to the FDA recommendation in scenario 2. However, similar to scenario 4, scenario 1 does not provide any information about prevalence in zone 1. Even at low prevalence, it is important to detect contamination in zones 1 and 2. In some scenario-time combinations, there was a negative slope, suggesting that the scheme identified fewer contaminated sites among a fixed number of samples compared to the overall proportion of contaminated sites in the facility (i.e., an underestimation of the true prevalence). Although this would provide confidence in perceived risks, the presence of undetected *Listeria* could pose a risk to consumers. As priorities shift, an operation may choose a combination of sampling schemes for different circumstances.

It is important to understand that “true prevalence” is defined here as the *Listeria* prevalence among the modeled agents in the operation. It does not include *Listeria* on the modeled floors, walls, or ceilings. One limitation of the model, and of all agent-based models, is that the number of agents is based on the discretion of the modeler founded on the best available understood relevance of the site to environmental monitoring. The complexity of a food facility complicates the task of defining every possible sampling site as an agent. This task is nearly impossible, and therefore not every site within the facility is modeled. Therefore, it is possible that key sites/agents were not included in the model, potentially resulting in an underestimation or overestimation of *Listeria* prevalence. This emphasizes the challenges of calculating true prevalence both *in silico* and in reality (as we cannot truly know the denominator). It is nearly impossible for a real (as opposed to modeled) facility to know the true prevalence and often impractical to estimate it.

### Influential parameters can be prioritized targets for corrective actions.

The probability of zone 4 introduction and the amount of *Listeria* introduced per object entering from zone 4 (e.g., carts or bins that move in and out of the room) were found to be influential parameters for the presence of *Listeria* in all zones in both facilities. Zone 4 sites are typically described as early indicators of the presence of potential food safety hazards (25). Additionally, several studies have detected *Listeria* in zone 4 sites and on movable objects, such as carts and trash bins, that potentially move from zone 4 into the production room (15, 26, 27). It is important to note that in a given operation, there is not always a well-defined barrier around the production area (e.g., wall) to truly separate zones 3 and 4. One study showed that interventions targeted at reducing zone 4 introduction, including door foamers and controlled movement of people and equipment into and out of finished product areas, had a considerable effect on reducing *Listeria* prevalence (23). Therefore, for the two facilities modeled here, these types of intervention strategies may be worth considering during a review of mechanisms driving the introduction of *Listeria* into the production environment.

The concentration of *Listeria* per contaminated incoming produce item was also consistently influential on the presence of *Listeria* across agents in all zones and in both facilities modeled. This is consistent with findings from a similar model of a cold-smoked salmon facility (9), which found this parameter (*N_R_*) to be influential, but found overall that the prevalence of *Listeria* in incoming product (*R_d_*) was the most significant input parameter for zone 2 *Listeria* prevalence. *Listeria* is present in the natural environment (28) and can therefore contaminate raw food products before entering a food processing facility. Contamination of raw product has been observed previously, such as with smoked fish (23, 29). However, limited information is available on *Listeria* prevalence in raw produce. Therefore, given the significant influence of contaminated raw product on the modeled *Listeria* prevalence in fresh-cut produce facilities, further studies are needed to better understand the *Listeria* prevalence of fresh produce entering a facility.

Although the two models had similar significant input parameters, the model for facility B had more statistically significant input parameters than that for facility A for all zones. One potential limitation is that some significant input parameters in this study showed partial rank correlation coefficients of less than 0.4, which may indicate that although the parameters are statistically significant, the magnitude of influence on the outcome (e.g., the presence of *Listeria* at the end of the shift) is likely not meaningful. Although there is no straightforward interpretation, influential parameters can be used to inform reviews of good manufacturing practices that affect *Listeria* prevalence, potentially helping to prioritize targets for corrective actions.

In closing, synthetic data generated via simulation of *Listeria* dynamics in two modeled fresh-cut produce facilities showed, through cluster analysis, that site-specific attributes impact the likelihood of contamination and therefore necessitate different sampling and surveillance schemes. Thus, models such as those developed here could be particularly useful as tools to support decision-making for fresh-cut produce facilities during the design and refining of sampling plans as part of EMPs. It is nearly impossible for facility personnel in the produce industry to try out and compare every sampling scheme. They need a practical approach that is science based to determine what approach may be most effective for their specific situation. Modeling can help with this. Further exploration of model-based approaches is important, as each time a computer model is created to elucidate a problem, learnings from previous models are incorporated. As model-based approaches continue to develop, simulations of real-world situations will improve, therein allowing for rapid scenario testing rather than spending days, months, or years in trial-and-error field testing. This will ultimately allow industry to make better decisions, such as how to detect potential issues quicker and with fewer samples and resources.

## MATERIALS AND METHODS

### Facility description and historical data.

Two models were created for this study, each representing a real fresh-cut produce facility. The facilities were selected from participants in a previous study, where 1 year’s worth of environmental sampling data was available (21). To receive permission to conduct research in these facilities, we agreed with the participating companies that we would keep the specifics of what produce commodities were processed in these facilities confidential to protect the company’s proprietary information.

Facility A has two rooms that are separated by a half wall with a plastic curtain. The product is brought into a raw receiving room (not modeled) where there is a bin dump area for the product to be transferred into a flume. The flume moves the product into the main production room through a window, where it is physically modified in some way (e.g., sliced), washed, dried, weighed, and packed. The main flume diverges into two washing lines and four weighing and packing lines. After product is packed into its primary packaging, it is moved to a separate room (not modeled) through windows for final packaging and storage or shipping. Facility A is a 24-h plant that has two production shifts and one sanitation shift Monday to Friday. A dry cleaning occurs after Friday’s second shift, and a deep clean begins Sunday evening. All three shifts have two 30-min breaks. Facility A processes approximately 4,400 lb of product/h. The temperature of the production rooms is kept between 34 and 60°F, with variation depending on time of day, seasonality, and number of people working. Facility A has between 30 and 40 people working during the production shifts and 5 to 10 working during sanitation.

Facility B has one production room. Product is brought into a raw receiving room (not modeled) where there is a bin dump area for the product to be transferred onto belts for presort before being transferred into flumes. The flumes or belts move the product into the main production room through doors, where the product is physically modified in some way (e.g., sliced), washed, dried, weighed, and packed. Facility B has seven flumes and washing lines that diverge into 10 weighing and packing lines. After the product is packed into its primary and secondary packaging, it is moved to a separate room (not modeled) for final packing and storage or shipping. There are two production shifts and one sanitation shift daily on Monday to Friday. On Saturday, there is one production shift, followed by dry cleaning. A deep cleaning shift begins Sunday evening. Three fewer flume and wash lines are used during the second shift. Approximately 67,000 and 40,000 lb are processed per hour during the first and second shifts, respectively. There are between 145 and 155 employees during the first shift, 60 to 70 during the second shift, and 25 during sanitation. The temperature of the main production room is approximately 36°F, with variation depending on seasonality and number of people working.

Historical data on microbiological testing for *Listeria* presence in both facilities were collected as part of a complementary study (21). Briefly, sites in zones 2 and 3 were sampled using individually packaged sponges hydrated with 10 ml D/E (Dey and Engley) neutralizing buffer (3M, St. Paul, MN). The sponge samples were collected at least 3 to 4 h into production. Sponge samples were analyzed to determine the presence of *Listeria* by use of the FDA “BAM method” as detailed in chapter 10 of the *Bacteriological Analytic Manual* (30).

### Modeling approach and implementation.

Agent-based models were used to recreate in detail the processing environments *in silico*. Modeled components were specific and unique to each facility and were developed based on in-person observations and information received from facility personnel. The floorplan of each facility was represented by a grid of squares, scaled to the size of the production area (3,854 squares for facility A and 8,256 squares for facility B). The modeled production environment included all walls, doors, mezzanines, and the floor in the facility blueprint (model interface shown in Fig. 1). The scale of each square (referred to as a patch) was dependent on the size of the operation. For facility A, each patch represented a 30- by 30-cm area. For facility B, each patch represented a 50- by 50-cm area. An identical grid of ceiling patches was also included and was located in a parallel plane at the height of the modeled rooms. Patch-specific traffic and water levels were dynamic and were imported on the floorplan grid during simulated production depending on the current shift or event (e.g., higher traffic during shift changes and breaks). Based on the shift or event, each patch had designations for both water and traffic levels of negligible, low, or high. The equipment and employees of each facility were represented as agents, containing unique and specific information including name, *x* and *y* coordinates in the model’s two-dimensional plane, zone category, distance from the floor, surface area, and whether or not the site was cleanable during routine sanitation. Zone category was determined based on a given agent’s proximity to food, with zone 1 being food contact surfaces (e.g., flume), zone 2 being directly adjacent to food contact surfaces (e.g., equipment frame), and zone 3 being in the product room where product is exposed but not directly adjacent to a food contact surface (e.g., drains, equipment wheels, door frames, pallet jacks, trash bins, conveyors, squeegees, ladders). No agents within these models were considered zone 4, which is outside of the exposed product areas (e.g., break room). However, introduction of *Listeria* from zone 4 was a submodel with input parameters specific to the produce facilities.

Facility A had 280 agents, while facility B had 698 modeled agents (Table 1). The distance from the floor of each agent represented its vertical location within the facility. Therefore, if an employee or piece of equipment was located on a mezzanine, then the distance from the floor included the height of the mezzanine. Mezzanines were represented as a patch layer located between the floor and ceiling patches at the height of the mezzanine. Therefore, in the two-dimensional (2D) interface that allows for modeling of 3D space, there were layers of spatial information for agents and the environment. For example, at a given *x,y* coordinate, there could be (from low to high) a floor patch, an agent, a mezzanine patch, another agent, and a ceiling patch. Connections between agents were represented by links and provided a network for potential *Listeria* cross-contamination in the processing facility. There were two kinds of links: directed and undirected. The directed link between agents represented a relationship where *Listeria* could transfer in a single direction between two agents (i.e., in-directed or out-directed). The undirected link represented a relationship where *Listeria* transfers in either direction between the two agents. The determination of links relevant to a given agent was done based on in-person observations if not already known. Facility-specific hourly events (i.e., empty, preproduction, first shift production, second shift production, dry clean, clean, empty) were simulated on an hourly time step according to each facility’s weekly production schedule.

The model algorithm (code) was written in the open source program NetLogo 6.1.1 (31) and is available on GitHub (https://github.com/IvanekLab/CPS_2019_OpenData). The code was adapted for this study from a previously developed agent-based model of a food facility, EnABLe (9), by tailoring the code to the specifics of each of the two facilities. Briefly, the setup of the *in silico* facility environment involved the importing of the agents and agent attributes, links, events, and maps. To initialize the model world, the initial environment was set to 0% *Listeria* prevalence among agents and patches. The start of the 2-week simulation began on Sunday at 12:01 a.m. The simulation start time does not affect outcomes and can be modified if desired by users. Throughout the week, *Listeria* was introduced into the environment via three ways: (i) contaminated raw product, (ii) zone 4 cross-contamination, and (iii) random events (i.e., unexpected introductions). The number of employees in the production room was predetermined and based on the time of day (and therefore the shift). *Listeria* was spread throughout the agents and patches in three ways: (i) patch-to-patch spread, (ii) agent-to-agent spread through the directed or undirected links, and (iii) agent-to-patch spread. Patch-to-patch spread occurred only if water or traffic levels in the area of patches were greater than negligible (i.e., low or high) at a given hour of the day. Condensation was also included in the model, with the possibility that *Listeria* can be transferred from the mezzanine underside or ceiling to the agents or patches directly below. *Listeria* growth and survival were dependent on the water level. *Listeria* removal from agents and patches occurred via transfer events and during modeled routine cleaning and sanitation, which resulted in a reduction in the concentration of any *Listeria* present in accordance with the sanitation log reduction parameter. The maximum specific growth rate of *Listeria* on environmental surfaces was determined using an equation from Giménez et al. (32) (Table 2). It should be noted that we are modeling growth of *Listeria* spp. on equipment and environmental surfaces, not in food products. We use studies of *Listeria* in produce to estimate these parameter values, under the assumption that *Listeria* requires the presence of moisture and organic matter (e.g., residual product) to grow on a surface. The generation time (GT) was calculated using values taken from the reported growth over time presented in a report by Ziegler et al. (33). This parameter was used to calculate the maximum specific growth rate (μ) for an iteration. The uniform distribution with the minimum and maximum GT provided a range of growth rates to evaluate across model iterations. The values used are in line with data presented in other references (34, 35).

### Input parameters.

The input parameters for the models were determined using either (i) in-person observations, (ii) published literature, or (iii) an expert elicitation (Table 2; see Table S1 in the supplemental material). Input parameters from published literature may be from facilities and commodities similar to but not identical to those modeled. This represents a pragmatic approach for development of ABMs that can be used for initial decision-making and sensitivity analyses that will allow for prioritization of further data collection efforts. For some input parameters, such as the proportion of *Listeria* transferred to an equipment surface upon contact with a contaminated product (α), there is an opportunity for selecting values more likely to align with fresh produce commodities. However, as this parameter was not found to be significant during sensitivity analysis, better data would not affect conclusions. Similarly, for the probability of *Listeria* species transfer (τ*ij*) between surfaces, published literature was used to understand the potential for *Listeria* transmission across zones (and therefore not between specific pieces of equipment), so different surface types and transmission routes were considered. In some instances, select parameters were assumed when other information was unavailable and was determined based on prior knowledge and experience. The expert elicitation was used for the estimation of five of the input parameters as well as the agent contact probabilities. The approach is often used to support evidence from empirical studies. For example, expert elicitation has been used for that purpose in risk assessments (36) and modeling (37). A strength of expert elicitation during early stages of novel modeling framework development, such as in this study, is that it permits rapid evaluation of the system and parameter uncertainty (37). Additionally, empirical estimation of the contact probability parameters (Table S2) would have required repeated observation (in person or by video) in the modeled facilities, which may be considered disruptive or intrusive by the facilities’ management and employees. Thus, a survey used for the expert elicitation was adapted from a survey used in our previous study (9), with updates made to include scenarios specific to fresh-cut produce. The survey was sent to and completed by six people that had expertise on *Listeria* in food facilities: four experts that work in academia and two experts that work in the produce industry. Each question in the survey targeted a specific parameter, with the median, minimum, and maximum result for each parameter being used for the relevant distribution. The reliance on expert opinion for initial parameter estimates represents a possible weakness, but in line with a report by Russell et al. in 2017 (37), it represents a viable approach, as it allowed us to use sensitivity analysis to determine for which parameters ground truth input parameters are most important and should be prioritized for future collection. This approach is essential to facilitate real-world use and implementation of ABMs in industry, as *a priori* collection of facility-specific data for all input parameters will represent an insurmountable hurdle to model implementation for industry.

### Verification and validation.

Several methods were used in verification steps when building the models, including syntax checking, visual testing, print statements, spot tests with agent monitors, code reviews, parameter validity, and different seed generators. Predictive validation was used for model validation and was done using historical microbiological data collected during a previous study (21). The predictive validation also included 52 additional samples that were collected most recently as part of this project with the intention of generating enumeration data. However, all additional samples were negative for *Listeria*. Model-simulated environmental sampling during production was consistent with the sample collection of the historical data by aiming to mimic the sampling site list from historical sampling with sampling sites in simulated sampling. The *Listeria* prevalence of the historical samples was compared with the average prevalence of samples collected during the second week of the simulation from the corresponding *in silico* sampling sites from 10,000 iterations of 2-week simulations in each facility model. As zone 1 sites were not sampled in historical data, their contamination status could not be explicitly validated using predictive validation. However, other modes of model verification described above were performed to verify that part of the model.

### Simulation and sensitivity analysis.

To assess the *Listeria* dynamics within the modeled facilities, simulation analyses were performed for the validated models. Multiple simulation iterations (i.e., trials) were completed (*n* = 10,000), generating simulated data that allowed for characterization of the *Listeria* prevalence on each zone during four phases of production: preop (hour prior to production), beginning of shift (first hour of production), midproduction (ninth hour of production), and end of day (18th hour of production).

Sensitivity analysis of validated models was performed using R Studio (version 1.2), using the prcc() command (38, 39) to determine the partial rank correlation coefficients to identify which of 53 key parameters (Table 2; Table S1) had a significant effect on *Listeria* prevalence by zone. Tornado plots of the significant parameters after Bonferroni’s correction (*P* < 0.05/53) were used to visualize the extent and directionality of the parameter influence on the outcome of interest. The code used for these analyses is available on GitHub (https://github.com/IvanekLab/CPS_2019_OpenData).

### Cluster analysis.

Hierarchical cluster analysis was used to group agents based on 10 continuous outcomes related to predicted *Listeria* contamination status: average *Listeria* concentration given contaminated, median time contaminated, maximum time contaminated, median number of contacts, median number of transfers, median number of temporary niches, median number of times a niche is contaminated, median number of times contaminated from incoming product, median number of times contaminated from zone 4 objects, and median number of times contaminated from a random event. A principal-component analysis (PCA) was performed using the FactoMineR package in R Studio (version 1.2) (40), with the number of dimensions determined based on a minimum cumulative variance explained of 80%. Hierarchical clustering on principal components was done using the same package in R Studio. The code used for these analyses is available on GitHub (https://github.com/IvanekLab/CPS_2019_OpenData).

### Scenario analysis.

The models were used to conduct scenario analyses to evaluate environmental monitoring programs and determine how sampling plans differ given five sampling schemes and three time points (Table 6). Each scenario was repeated for 1,000 iterations with a fixed seed to ensure that the same combinations of input parameter values were used for each successive iteration across all 15 scheme-time combinations. The first scenario was the baseline and represented the sampling program currently conducted by the facility during routine sampling. The second scenario represented the FDA *Listeria* draft guidance recommendation (41), which specifies that at least 5 samples should be collected per line on food contact surfaces and 5 samples per line on nonfood contact surfaces. The third scenario represented a completely random sample collection, where all agents in the facility had an equal chance of being selected for sampling, regardless of zone category. For the fourth scenario, simulated sampling was collected only from zone 3 agents. The fifth scenario represented sites selected based on the cluster analysis of model predictions and thus targeted sites with characteristics conducive to becoming and remaining contaminated. All scenarios were simulated at three different sample collection times on Wednesday of the second week of the 2-week simulation: (i) the first hour of production, (ii) the fourth hour into production (as is recommended by the FDA [41]), and (iii) the tenth hour into production. Sampling performance was calculated as the proportion of *Listeria*-contaminated agents detected among all those sampled minus the true prevalence at the same time point (i.e., the proportion of contaminated agents among all modeled agents):

$$\text{sampling performance}=\text{proportion sampled positive}-\text{true prevalence}=\left(\frac{x}{s}-\frac{y}{n}\right)\times 100$$

where *s* is the number of agents sampled in a given sampling scheme out of *n* possible sampling sites (i.e., number of agents in the facility), *x* is the number of sampled agents that tested positive, and *y* is the total number of contaminated agents in the facility. The sampling performance was then plotted against true prevalence to visualize the stability over a range of contamination levels in the facility. The code used for these analyses is available on GitHub (https://github.com/IvanekLab/CPS_2019_OpenData).

### Data availability.

Data files and code used to build the two agent-based models using NetLogo, as well as data files and R code relevant to the cluster analysis, scenario analysis, and sensitivity analysis, are available on GitHub at https://github.com/IvanekLab/CPS_2019_OpenData.
