# Supplementary material for: In Silico Models for Design and Optimization of Science-Based Listeria Environmental Monitoring Programs in Fresh-Cut Produce Facilities
Source: Appl Environ Microbiol. 2021 Oct 14;87(21):e00799-21. doi: 10.1128/AEM.00799-21 (PMC8516048; doi:10.1128/AEM.00799-21)
Supplement: Supplemental file 1 — Tables S1 to S3. Download AEM.00799-21-s0001.pdf, PDF file, 0.1 MB [file aem.00799-21-s0001.pdf]

Table S1. Model parameters, distribution information, values, and sources for *Listeria* transmission between agents.

Symbol	Description [a]	Distribution	Mean	5th-95th percentile	Reference
p11	Probability of contact from contaminated surface in Zone 1 to another surface in Zone 1	Pert (0.0, 0.10, 0.90, 4)	0.22	[0.30, 0.49]	expert opinion
p12	Probability of contact from contaminated Zone 1 to Zone 2	Pert (0.00, 0.20, 0.80, 4)	0.27	[0.61, 0.52]	expert opinion
p13	Probability of contact from contaminated Zone 1 to Zone 3	Pert (0.00, 0.15, 0.85, 4)	0.24	[0.48, 0.53]	expert opinion
p14	Probability of contact from contaminated Zone 1 to an employee	Pert (0.00, 0.10, 0.80, 4)	0.20	[0.28, 0.45]	expert opinion
p21	Probability of contact from contaminated Zone 2 to Zone 1	Pert (0.00, 0.20, 0.95, 4)	0.29	[0.56, 0.60]	expert opinion
p22	Probability of contact from contaminated Zone 2 to another Zone 2	Pert (0.00, 0.15, 0.70, 4)	0.22	[0.44, 0.44]	expert opinion
p23	Probability of contact from contaminated Zone 2 to Zone 3	Pert (0.00, 0.20, 0.85, 4)	0.28	[0.62, 0.55]	expert opinion
p24	Probability of contact from contaminated Zone 2 to an employee	Pert (0.00, 0.10, 0.80, 4)	0.20	[0.35, 0.44]	expert opinion
p31	Probability of contact from contaminated Zone 3 to Zone 1	Pert (0.00, 0.2, 0.90, 4)	0.16	[0.01, 0.40]	expert opinion
p32	Probability of contact from contaminated Zone 3 to Zone 2	Pert (0.00, 0.04, 0.90, 4)	0.17	[0.01, 0.43]	expert opinion
p33	Probability of contact from contaminated Zone 3 to another Zone 3	Pert (0.00, 0.13, 0.85, 4)	0.23	[0.04, 0.49]	expert opinion
p34	Probability of contact from contaminated Zone 3 to an employee	Pert (0.00, 0.02, 0.60, 4)	0.11	[0.01, 0.29]	expert opinion
p41	Probability of contact from contaminated employee to Zone 1	Pert (0.00, 0.10, 0.95, 4)	0.23	[0.03, 0.51]	expert opinion
p42	Probability of contact from contaminated employee to Zone 2	Pert (0.00, 0.10, 0.80, 4)	0.20	[0.02, 0.45]	expert opinion
p43	Probability of contact from contaminated employee to Zone 3	Pert (0.00, 0.10, 0.90, 4)	0.22	[0.03, 0.51]	expert opinion
p44	Probability of contact from contaminated employee to another employee	Pert (0.00, 0.05, 0.40, 4)	10	[0.01, 0.22]	expert opinion

[a] All parameter values correspond to an hourly time-scale, the time-scale of the model.

Table S2. Transfer coefficient matrix based on zone or employee type. Transfer coefficient was selected based on a combination of the sender (i: horizontal) and receiver (j: vertical). Values are log10 of the transfer coefficient.

	Zone 1	Zone 2	Zone 3	Employee
Zone 1	-1.51	-3.68	-0.28	-1.97
Zone 2	-1.51	-1.51	-3.53	-1.97
Zone 3	-0.31	-0.31	-0.31	-0.82
Employee	-1.97	-1.97	-1.69	-3.43

Table S3. Standard deviation matrix based on zone or employee type. Standard Deviation was selected based on a combination of the sender (i: horizontal) and receiver (j: vertical).

	Zone 1	Zone 2	Zone 3	Employee
Zone 1	0.2	0.2	0.2	0.87
Zone 2	0.2	0.2	0.2	0.87
Zone 3	0.2	0.2	0.2	0.87
Employee	0.87	0.87	0.87	0.79
